# The epidemiology of infectious mononucleosis in Northern Scotland: a decreasing incidence and winter peak

**DOI:** 10.1186/1471-2334-14-151

**Published:** 2014-03-20

**Authors:** Elizabeth Visser, Denis Milne, Ian Collacott, David McLernon, Carl Counsell, Mark Vickers

**Affiliations:** 1 Division of Applied Health Sciences, College of Life Sciences and Medicine, University of Aberdeen, Room 1:015, Polwarth Building, Foresterhill, Aberdeen AB252ZD, UK; 2 Department of Haematology, Aberdeen Royal Infirmary, Foresterhill, Aberdeen AB25 2ZN, UK; 3 Department of Virology, Aberdeen Royal Infirmary, Foresterhill, Aberdeen AB25 2ZN, UK; 4 Division of Applied Health Sciences, University of Aberdeen, Polwarth Building, Foresterhill, Aberdeen AB25 2ZD, UK; 5 College of Life Sciences and Medicine, University of Aberdeen, Polwarth Building, Foresterhill, Aberdeen AB25 2ZD, UK

**Keywords:** Seasonality, Epstein-Barr virus (EBV), Infectious Mononucleosis (IM), Epidemiology

## Abstract

**Background:**

Infection with Epstein-Barr virus (EBV) is almost ubiquitous in humans and generally occurs at two ages: infantile, which is usually asymptomatic and associated with poorer socioeconomic conditions, and adolescent, which causes infectious mononucleosis (IM) in ~25% cases. The determinants of whether the infection causes IM remain uncertain. We aimed to evaluate seasonality and temporal trends in IM.

**Methods:**

Data from all Monospot tests, used as a marker for IM, were collected from the Grampian population over 16 years.

**Results:**

Positive Monospot test results peaked at 17 years in females and 19 in males. Females had 16% more diagnoses, although 55% more tests. IM was ~38% more common in winter than summer. The annual rate of positive tests decreased progressively over the study period, from 174/100 000 (95% CI 171–178) in 1997 to 67/100 000 (95% CI 65–69) in 2012.

**Conclusions:**

IM appears to be decreasing in incidence, which may be caused by changing environmental influences on immune systems. One such factor may be exposure to sunlight.

Words 168.

Funding The Medical Research Council and NHS Grampian-MS endowments.

## Background

The principle that diseases caused by infectious agents arise from complex interactions between host and pathogen is exemplified by the constellation of symptoms and complications that can arise from Epstein–Barr virus (EBV). EBV is a human-specific herpes virus that infects 90-95% of adults [1] and has co-evolved with its host. In non-industrialised countries, over 90% of primary infections occur in the first few years of life [2] and cause no distinct symptoms. In industrialised countries, probably because of higher standards of hygiene [3], 25-40% of children escape primary infection [4]. Instead, infection is delayed until adolescence or early adulthood, when about 25% of infections cause infectious mononucleosis (IM) [5], which is variable in its presentation but commonly manifests as pharyngitis, fever and lymphadenopathy [1]. The immune response in IM is unusually strong [6] and is thought to underlie the severity of the disease.

After primary infection, the virus establishes a latent infection in memory B-lymphocytes [7]. The virus has transforming properties and may later cause serious diseases such as Hodgkin lymphoma, several forms of non-Hodgkin lymphoma or nasopharyngeal carcinoma [8]. It has also been associated with chronic fatigue syndromes and an increased risk for immune mediated diseases such as multiple sclerosis (MS) in later life, although causality is uncertain [9-12].

Although genetic influences encoded within both class I and II major histocompatibility complex loci and mode of infection are thought to determine whether individuals develop IM [13], why the immune response to this infectious agent changes profoundly with respect to age remains poorly understood. Age dependent processes such as variation in host antibodies and pre-existing infection resulting in cross-reactions in T-cell populations may play important roles [14,15].

In addition to age, there are numerous environmental influences on immune responses. Increases in the incidences of allergic and autoimmune diseases over the last 50 years are well described [16,17]. The reasons for these increases are uncertain, although declines in infectious diseases have been suggested. One environmental factor that has attracted much recent attention is vitamin D. Vitamin D levels are determined by both oral intake and exposure to sunlight, so that vitamin D levels are higher in summer than winter. Seasonality in both infectious and non-infectious diseases has been described as far back as Hippocrates (~380 BC) [18]. Presentation of several immune mediated diseases is higher in spring and in those who were born in spring [18-20]. Receptors for vitamin D are widely expressed in the immune system [21] and vitamin D has been shown to promote immune tolerance in dendritic antigen presenting cells [22].

We postulated that IM might be the result of a relative deficiency in immune tolerance. If infection occurs in the winter months, when vitamin D levels are lower, there might be a greater risk for developing infectious complications [23], especially IM [24]. In an epidemiological study of IM conducted in the 1960s in North-East Scotland no seasonal variation was demonstrated and the incidence was noted to be increasing, which may have reflected increasing awareness [25]. We performed a more extensive epidemiological survey of IM over a 16 year period, specifically looking for evidence of seasonality and changing rates of IM.

## Methods

### Tests

Data from patients who had a heterophile antibody test (Monogen rapid latex particle agglutination test, Monospot test) for acute EBV infection performed during the period from 1997–2012 were collected anonymously. The same test kit was used throughout the study period. The Monospot test sensitivity and specificity for acute EBV infection are both ~93% [26]. Serological EBV test data (EBV viral capsid antigen IgM test) for the period 2000 to 2012 were also gathered anonymously. The immunology laboratory moved from a combination of EIA (Enzyme Immuno-Assay) and immunofluorescence used up to 2012, to using EIA only from 2012 onwards.

### Study population

Clinical information on the onset of symptoms, indication for testing and demographic information on permanent or temporary residency in the area were not available. Age, gender, date of testing and results of tests were collected retrospectively from the haematology laboratory of Aberdeen Royal Infirmary, which is the sole provider of diagnostic services for the population of 570 526 in the Grampian area (latitude 56.837° N to 57.41°N) [27]. Tests received from both primary and secondary care were included. The Grampian area includes Aberdeen city, a number of smaller towns and large farming communities. There are a number of secondary and tertiary educational facilities that attract students from the UK and worldwide. According to the 2001 census, 6% of the population in this area were not native to Scotland and that figure recorded in 2011 is now 16% [28]. An enquiry to the North of Scotland Research ethics committee revealed that no ethics permission was needed for this project.

### Statistical analysis

Annual age-gender specific rates of positive Monospot tests were calculated using the relevant Grampian population as published by the Information Services Division (ISD) of Scotland [27] and then standardised against the June 2009 Scottish population [29]. Confidence intervals were calculated assuming a Poisson distribution. Microsoft Excel and StatsDirect were used to calculate trends, rates and confidence intervals. The numbers of serological tests for acute EBV infection were compared with Monospot tests to account for any change in diagnostic methods for IM. Median ages for males and females and interquartile ranges (IQR) were calculated and the difference in ages for males and females was analysed using a Mann–Whitney U non-parametric test.

Data on all tests and positive results were tested for seasonal trends using modified Roger’s and Edwards’ tests. The number of tests were plotted by month and a sinusoidal curve of best fit was included [30,31]. These analyses were repeated for different age groups and gender. The peak months, amplitudes (with crude 95% confidence intervals [CI]) and significance levels were calculated from the sinusoidal curves.

### Role of funding source

The funding sources had no role in the study design, data collection, analysis, interpretation or writing of the report. The corresponding author had final responsibility for the decision to submit for publication.

## Results

**Figure 1:**
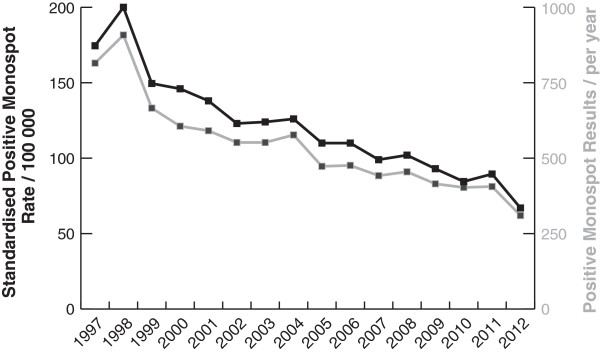
**Positive Monospot tests from 1997 to 2012.** Number of tests performed per year shown by grey line and right hand scale. Age-gender standardised rate shown by black line and left hand scale.

General population data for Grampian were collected bi-annually before 2001 and quarterly thereafter and showed no evidence of seasonal variation and change over time. The original database comprised IM test results of 62228 requests from January 1997 to December 2012 (See Additional file 1). We first plotted the number of positive Monospot tests with respect to time. A substantial downward trend was apparent, with the number of cases approximately halving over the study period (Figure 1). As the result was unexpected, we tested whether several confounding factors might explain the decrease. Initially, we tested for changes in the denominator population. Age-gender standardised rates of positive Monospot tests showed the same trend (Figure 1). The annual rates of positive IM tests declined from 174/100 000 (95% CI 171–178) in 1997 to 67/100 000 (95% CI 65–69) in 2012 (See Additional file 1 for data and populations).

**Figure 2:**
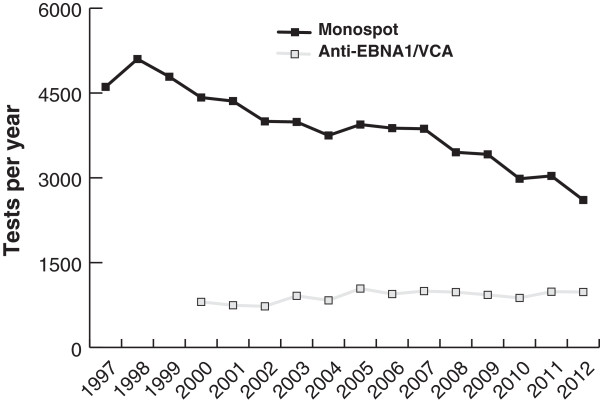
Total Monospot and EBV specific antibody tests performed 1997 to 2012.

We also considered whether tests for IM investigating EBV specific antibodies performed by the virology laboratory might have been increasingly favoured above the Monospot tests performed by the haematology laboratory. Serological data were available from 2000 onwards. There was an increase in test requests for EBV specific antibodies (n = 806 in 2000 to n = 981 in 2012) (Figure 2), but this could not account for most of the decrease in Monospot requests.

**Figure 3:**
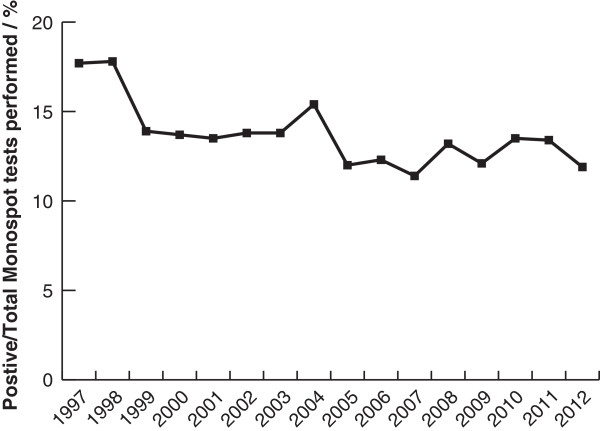
Positive / Total Monospot IM test results from 1997 to 2012.

Our third consideration was that ‘diagnostic fashion’ might have changed over the study period. For instance, patients might have visited their doctor less with symptoms of upper respiratory tract infection suggestive of IM or doctors might have become more selective in their use of confirmatory testing. In both cases, the fall in test requests should have been greater than the decrease in positive tests. Figure 3 shows this was not the case; the proportion of positive results decreased over the study period.

**Figure 4:**
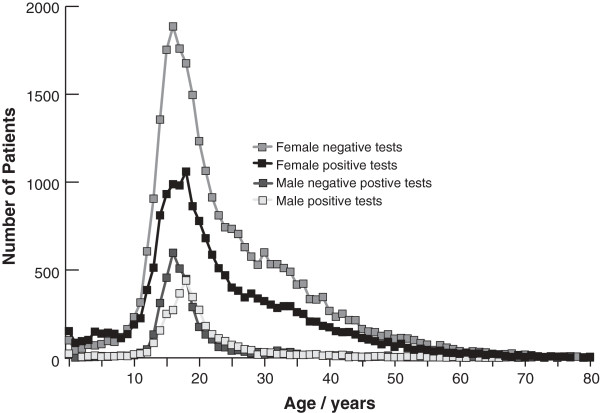
Number of positive and negative Monospot tests by age and gender.

We went on to analyse the ages and genders of tests and diagnoses, which revealed several features of interest. Figure 4 shows the characteristic adolescent peaks of diagnoses; the female median age was 17 [IQR 15–20] and the male median age 19 [IQR 16–22]. The female peak age is significantly (p < 0.0001) younger than the male. Furthermore, more females were diagnosed with the disease. However, the number of requests for females was considerably greater, perhaps because overall female consultation rates are higher than those of males. Analysis of temporal trends split by gender showed the decreases in both positive and negative tests were similar for both males and females (data not shown). Furthermore, diagnostic yield falls progressively after the age of 20 and is very low over the age of 30.

**Figure 5:**
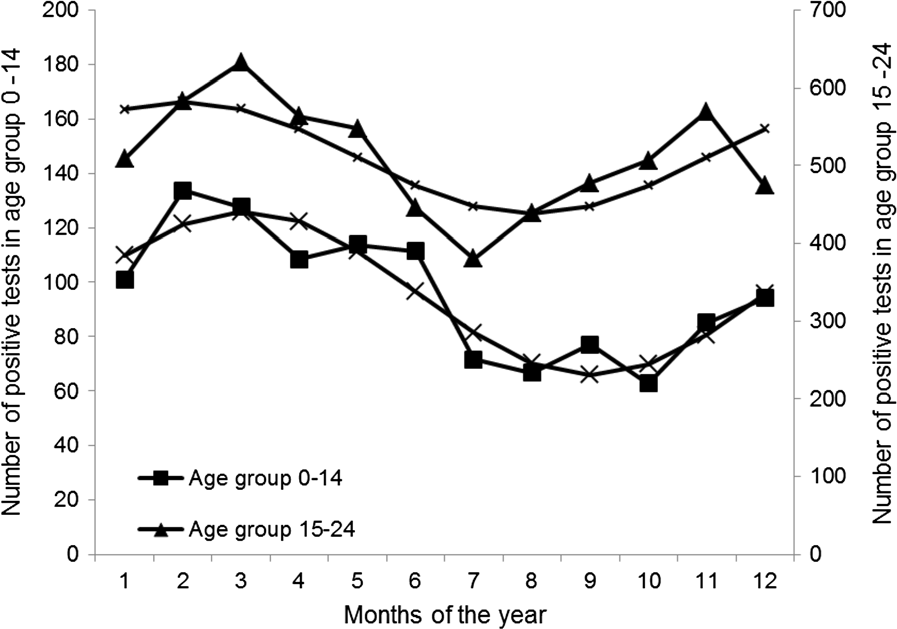
Seasonality of positive Monospot tests in age groups 0–14 and 15–24 years.

Two statistical tests, Edwards’ and Roger’s tests, for analysis of cyclical variation in tests were utilised to explore the hypothesis of seasonality in testing patterns and test results. The peak month, amplitude and 95% confidence intervals (CI) are displayed in Table 1 and significance expressed as p-values, where p < 0.001 is statistically significant, are also shown. The amplitude varied from 10.5% to 32.4% in the statistically significant groups. The number of positive tests was seasonal in all patients, men and women aged 0–24 years, the age range when a positive test is likely to be associated with IM. Overall, the peak rate of positive tests was recorded in February and trough rates in August with statistically significant seasonal variation. This late winter peak and summer trough pattern is replicated further after splitting the total number of positive tests by gender and by age groups of 0–14 years and 15–24 years. Figure 5 presents the seasonal trend of positive tests separately for these two age groups, with sinusoidal curves.

**Table 1:** Peak month, Amplitude and Significance of tests for seasonal trends in total and positive Monospot test results by age and gender

**Positive test results**					
	**Numbers analysed**	**Peak month**	**Amplitude (%)**	Crude 95% CI*	**Edwards’ & Roger’s P**
**All tests**					
**0-14**	1150	March	31.3	31.0-31.5	<0.0001
**15-24**	6119	February	14.3	14.2-14.3	<0.0001
**25-44**	1156	March	1.9	1.7-2.1	0.9
**>45**	222	August	18.3	17.0-19.6	0.3
**All ages**	8647	February	13.8	13.8-14	<0.0001
**Female**					
**0-14**	691	March	32.4	32.0-32.8	<0.0001
**15-24**	3300	February	16.2	16.1-16.3	<0.0001
**25-44**	554	February	8.1	7.6-8.6	0.1
**>45**	101	April	29.1	26.3-31.8	0.3
**All ages**	4646	February	16.6	16.6-17.0	<0.0001
**Male**					
**0-14**	459	March	29.9	29.3-30.5	<0.0001
**15-24**	2819	February	11.9	11.8-12.0	<0.0001
**25-44**	602	July	4.4	3.9-4.9	0.6
**>45**	121	August	20.3	18.1-22.7	0.2
**All ages**	4001	February	10.5	10.5-10.6	<0.0001
**Total tests performed**					
	**Numbers analysed**	**Peak month**	**Amplitude (%)**	Crude 95% CI*	**Edwards’ & Roger’s P**
**All tests**					
**0-14**	9884	February	34.5	34.4-34.5	<0.0001
**15-24**	30663	February	12.2	12.2-12.2	<0.0001
**25-44**	17615	April	12.9	12.9-12.9	<0.0001
**>45**	4066	March	13.0	12.9-13.1	<0.0001
**All ages**	62228	February	13.8	13.8-13.8	<0.0001
**Female**					
**0-14**	5565	February	32.1	32.0-32.2	<0.0001
**15-24**	18873	February	13.1	13.1-13.1	<0.0001
**25-44**	10936	March	16.6	16.5-16.6	<0.0001
**>45**	2449	March	17.0	16.9-17.1	<0.0001
**All ages**	37823	February	14.4	14.4-14.4	<0.0001
**Male**					
**0-14**	4319	February	37.6	37.5-37.7	<0.0001
**15-24**	11790	February	10.8	10.8-10.9	<0.0001
**25-44**	6679	April	6.9	6.9-7.0	0.009
**>45**	1617	March	7.5	7.4-7.7	0.1
**All ages**	24405	February	12.8	12.8-12.9	<0.0001

*CI: 95% Confidence intervals.

## Discussion

We performed a survey of the number of positive and negative IM test results in the Grampian region over 16 years, motivated by a desire to investigate seasonality. The major change was a decrease in the number of diagnoses over the study period. A seasonal variation was also detected, with the disease peaking in late winter.

The large number of cases and consistent trend makes us confident that our main finding is not a result of random variation. We also controlled for possible decreases in the number of susceptible individuals by analysing data about the size and age structure of the Grampian population over the study period so making changes in the denominator population an unlikely cause of the reduction in the IM rate. We are confident that we captured essentially all the diagnostic tests performed in our study area; all the General Practices in Grampian have a collection every weekday with delivery to a central laboratory. However, it is important to highlight that this was not set up as a formal incidence study on primary infection with EBV. In addition, changes in ‘diagnostic behaviour’ are difficult to control for. For instance, it is possible that the decrease may have arisen from either patients with IM seeking medical advice less often or doctors becoming less likely to request confirmatory tests over the last 15 years. All IM tests are provided without charge to both General Practitioners and hospital physicians, so there was no financial incentive to change behaviour. More objectively, other haematological blood tests from General Practitioners in Grampian have increased 2.2 fold over this time period.

The above findings are in contrast with the previous data from our study area, although there were significant methodological differences between the studies and the 1960s study also included patients for the Northern Islands of Scotland. Over the period of 1960–1969, 8828 cases were investigated and 1258 seropositive cases were found. The incidence increased from 11.1 to 44.3 cases per 100 000 and there was no significant seasonal variation in seropositive or seronegative cases [25]. Due to a number of different factors, of which the rise of the oil industry in this area is an example, the general population structure has also changed over time. Caution should therefore be used when comparing these studies.

Further evidence for variation in the prevalence of IM comes from other studies. In a survey of British General Practices incorporating a similar sized denominator population of 600 000, Morris and Edmunds [32] described a reduction in annual General Practitioner consultation rates for IM from 150–200 per 100 000 in 1970 to ~100 per 100 000 in 1999, with the decrease more marked towards the end of this period. Hospital admission data for which IM was listed as the primary diagnosis between 1997 and 2009 in our area showed decreasing numbers from 84 to 44 per year from 1997 to 1999, since when the number has remained stable (data not shown). This decline in the number of admissions is similar in magnitude to the decline in numbers of positive heterophile antibody results over the study period and contrasts with the report by Morris and Edmonds, where the number of admissions rose despite a fall in the number of test results. In a fourteen year surveillance study from France, Tattevin et al. described a significant increase in the annual incidence of sever Epstein-Barr virus related IM between 2002 and 2004. They reported on patients requiring admission to hospital admitted and although the numbers were small (n = 38) this is also in contrast to our findings [33].

We believe that the marked reduction we observed in positive Monospot tests reflects a genuine decrease in the number of cases of IM. IM is less common in countries with lower living standards [2] and thus presumably there was a previous increase in the rate of IM in Western countries as living standards improved, although it is uncertain when this occurred. If our data are confirmed by other surveys, it appears that this effect seems to be reversing at a relatively rapid rate, which, if sustained, would result in IM becoming a rare disease in approximately 15 years’ time.

This decline might be explained by a shift back to earlier infections [34,35] or a reduction in overall infections with EBV, so that more adults remain seronegative. However, in 2009 the Aberdeen clinical virology laboratory detected antibodies to EBV in 52, 71, 89, 96 and 96% of diagnostic samples analysed in the age groups <10, 10–19, 20–29, 30–39 and >40 respectively (n = 64, 202, 183, 137, 313). Two surveys in the nearby city of Edinburgh [3,5] showed that 56% of 11 year olds and 75% of matriculating undergraduates were seropositive. In a 2010 survey of pregnant mothers in Aberdeen only 5/273 were seronegative for EBV (unpublished data). All these data indicate that the overall rate of infection has not declined and late infection continues to affect about one third of individuals.

The reasons why a minority of individuals develop IM in response to adolescent primary infection with EBV remain unclear, but it is seems likely that Western immune systems have changed over the last few decades [16,17]. Environmental factors that could be driving these changes may also have decreased susceptibility to IM.

Our analysis also demonstrated seasonal variation, with IM being about a third more common in February than August. An incubation period of four to six weeks implies the peak of IM coincides with when vitamin D levels are at their lowest. While consistent with vitamin D deficiency being a possible contributory factor to the seasonality of IM, other explanations are possible. For instance, IM may be more common for the same, alternative reasons that infections with other viruses are more common in the winter months [36]. Splitting the seasonality analyses by age (Table 1) reveals that the effect is strongest at young ages, declines with increasing age and even reverses over the age of 45. Perhaps, therefore, term times may impact on seasonality [5]. However, to explain the increase in IM in winter, the influx of ‘at risk’ students returning to Grampian for winter vacation would have to greatly exceed the number of students leaving Grampian for their vacation and we were unable to find data to confirm this.

The documentation of the seasonality of EBV infection and specifically IM is historically inconsistent. In 1957 Newell described a seasonal pattern in IM patients older than 15 years of age and resident in London, with the incidence being higher in summer and autumn compared to winter and spring [37]. No seasonal pattern was recognised in a study from Rochester, Minnesota for the period of 1950 to 1969 [38] and in another study conducted during the period of 1969 to 1970 in colleges and universities in the United States of America, no consistent seasonal variation for IM was apparent [39]. A seasonal pattern of EBV infection was described in 1972 in Atlanta, Georgia. This showed two peaks in January (late winter) and September or October (early autumn) [40]. A peak in IM admissions to the Boston Royal Infirmary was found in a 12 year study in the month of October [41]. Chang et al. reported that season did not influence the rate of EBV seroconversion in children in a nursery [42]. In a large group, aged 18–23 from the Israeli Defence Force, cases of IM were recorded from 1988 to 1991. However, the peak incidence for IM was recorded during the summer months of June through to August [43]. The results from this body of literature are difficult to compare due to methodological and population differences. Overall, the hypothesis that lower levels of Vitamin D may be contributing to the higher infection rate during the winter months, an effect that may well be more marked at high latitudes, remains a possibility, but cannot explain all these discrepant data.

In addition to the main two observations of the study, one other feature of our data merits comment. Our survey reported the number of positive Monospot tests was 16% higher in females. This is in keeping with findings from a previous study in university students in Edinburgh where a higher female EBV seropositivity prevalence rate was found [44]. However, in our study the request rate for females was ~55% more than males, with a correspondingly higher number of negative test results. Females are known to visit doctors more often than men and it is likely that the number of true cases of IM may well then be similar with respect to gender [45]. Our data suggest that the rate of acute EBV infection seems to be declining at a similar rate for both males and females.

At present, the most common test used for diagnosing IM in the Grampian laboratory remains the Monospot test (Figure 2). Literature suggests that the sensitivity of the Monospot test can be as low as 85% and false positives can be seen with other infections [1,46]. The alternative is serological testing. Data on age and gender rates for serological testing were not available for this project; this remains a question for future research informing this epidemiological study.

## Conclusion

We performed a survey of the rates of positive Monospot tests in Grampian, which halved over the 16 year period and contrasts with previous increases in the study area. This decrease might be caused by changing environmental influences on immune responses. In addition, we found a higher frequency of IM in late winter, in accordance with the hypothesis that lower vitamin D levels might help cause IM.

## Abbreviations

EBV: Epstein-Barr virus; IM: Infectious mononucleosis.

## Competing interests

We are not aware of any conflicts of interest for any of the authors.

## Authors’ contribution

EMV and MAV were the principal investigators of this project and both worked on the literature searches, data interpretation, figures and writing of the manuscript. CEC provided advice and epidemiological expertise. DM and IC collated the data from the haematology and virology laboratories. DJMc helped EMV analyse and interpret the data and provided statistical expertise for seasonality analysis. EMV wrote the first draft and all the authors contributed to the final manuscript. All authors read and approved the final manuscript.

## Pre-publication history

The pre-publication history for this paper can be accessed here:

http://www.biomedcentral.com/1471-2334/14/151/prepub

## Supplementary Material

Additional file 1: Table S1 Negative Monospot test results by year, month age and gender. **Table S2.** Positive Monospot test results by year, month age and gender. **Table S3.** Population by year, age and gender from Grampian ISD data [27]. **Table S4.** Population for the June 2009 Scottish population by age and gender used for standardisation [29]. Click here for file
