# Supplementary material for: The epidemiology of infectious mononucleosis in Northern Scotland: a decreasing incidence and winter peak
Source: BMC Infect Dis. 2014 Mar 20;14:151. doi: 10.1186/1471-2334-14-151 (PMC3999936; doi:10.1186/1471-2334-14-151)
Supplement: Additional file 1: Table S1 — Negative Monospot test results by year, month age and gender. Table S2. Positive Monospot test results by year, month age and gender. Table S3. Population by year, age and gender from Grampian ISD data [27]. Table S4. Population for the June 2009 Scottish population by age and gender used for standardisation [29]. [file 1471-2334-14-151-S1.docx]

**The Epidemiology of Infectious Mononucleosis in Northern Scotland: a Decreasing Incidence and Winter Peak**

**Additional information**

**Table 1: Negative Monospot test results by year, month age and gender**

|  |  | **Negative** |  |  |  |  |  |  |  | |  | |  | |  |
| --- | --- | --- | --- | --- | --- | --- | --- | --- | --- | --- | --- | --- | --- | --- | --- |
|  |  | **Female** |  |  |  |  | **Male** |  |  | |  | |  | |  |
|  | **Age** | 0-14 | 15-24 | 25-44 | 45+ | Total | 0-14 | 15-24 | | 25-44 | | 45+ | | Total | **Total** |
| **Year** | 1997 | 426 | 1131 | 673 | 102 | 2332 | 318 | 632 | | 432 | | 80 | | 1462 | 3794 |
| **Month** | 1 | 45 | 103 | 47 | 8 | 203 | 28 | 54 | | 33 | | 4 | | 119 | 322 |
|  | 2 | 46 | 127 | 65 | 12 | 250 | 34 | 70 | | 29 | | 4 | | 137 | 387 |
|  | 3 | 51 | 121 | 67 | 16 | 255 | 49 | 67 | | 30 | | 10 | | 156 | 411 |
|  | 4 | 33 | 86 | 57 | 9 | 185 | 29 | 51 | | 48 | | 2 | | 130 | 315 |
|  | 5 | 37 | 97 | 70 | 14 | 218 | 32 | 43 | | 35 | | 6 | | 116 | 334 |
|  | 6 | 34 | 76 | 57 | 8 | 175 | 30 | 49 | | 45 | | 6 | | 130 | 305 |
|  | 7 | 19 | 67 | 50 | 6 | 142 | 20 | 39 | | 27 | | 8 | | 94 | 236 |
|  | 8 | 17 | 70 | 36 | 5 | 128 | 11 | 41 | | 36 | | 4 | | 92 | 220 |
|  | 9 | 39 | 95 | 49 | 5 | 188 | 10 | 46 | | 37 | | 6 | | 99 | 287 |
|  | 10 | 30 | 93 | 61 | 7 | 191 | 22 | 66 | | 41 | | 14 | | 143 | 334 |
|  | 11 | 40 | 101 | 54 | 5 | 200 | 24 | 58 | | 41 | | 10 | | 133 | 333 |
|  | 12 | 35 | 95 | 60 | 7 | 197 | 29 | 48 | | 30 | | 6 | | 113 | 310 |
| **Year** | 1998 | 405 | 1219 | 779 | 140 | 2543 | 356 | 747 | | 467 | | 81 | | 1651 | 4194 |
| **Month** | 1 | 39 | 111 | 70 | 7 | 227 | 20 | 74 | | 51 | | 3 | | 148 | 375 |
|  | 2 | 53 | 101 | 58 | 12 | 224 | 49 | 72 | | 46 | | 2 | | 169 | 393 |
|  | 3 | 40 | 123 | 71 | 13 | 247 | 47 | 58 | | 31 | | 9 | | 145 | 392 |
|  | 4 | 26 | 103 | 66 | 9 | 204 | 30 | 63 | | 41 | | 7 | | 141 | 345 |
|  | 5 | 45 | 79 | 68 | 17 | 209 | 33 | 47 | | 42 | | 10 | | 132 | 341 |
|  | 6 | 39 | 98 | 89 | 20 | 246 | 37 | 55 | | 44 | | 5 | | 141 | 387 |
|  | 7 | 27 | 83 | 80 | 18 | 208 | 19 | 52 | | 35 | | 18 | | 124 | 332 |
|  | 8 | 17 | 99 | 56 | 8 | 180 | 14 | 63 | | 36 | | 6 | | 119 | 299 |
|  | 9 | 29 | 113 | 70 | 10 | 222 | 22 | 69 | | 43 | | 3 | | 137 | 359 |
|  | 10 | 24 | 90 | 47 | 11 | 172 | 20 | 64 | | 34 | | 2 | | 120 | 292 |
|  | 11 | 42 | 139 | 52 | 7 | 240 | 35 | 65 | | 37 | | 6 | | 143 | 383 |
|  | 12 | 24 | 80 | 52 | 8 | 164 | 30 | 65 | | 27 | | 10 | | 132 | 296 |
| **Year** | 1999 | 359 | 1174 | 788 | 125 | 2446 | 314 | 780 | | 465 | | 118 | | 1677 | 4123 |
| **Month** | 1 | 39 | 87 | 74 | 12 | 212 | 29 | 48 | | 46 | | 14 | | 137 | 349 |
|  | 2 | 33 | 98 | 68 | 11 | 210 | 39 | 67 | | 35 | | 16 | | 157 | 367 |
|  | 3 | 68 | 141 | 80 | 17 | 306 | 33 | 105 | | 51 | | 9 | | 198 | 504 |
|  | 4 | 34 | 89 | 78 | 11 | 212 | 38 | 69 | | 56 | | 10 | | 173 | 385 |
|  | 5 | 23 | 78 | 64 | 12 | 177 | 22 | 50 | | 39 | | 5 | | 116 | 293 |
|  | 6 | 28 | 95 | 56 | 13 | 192 | 39 | 49 | | 29 | | 8 | | 125 | 317 |
|  | 7 | 21 | 85 | 52 | 7 | 165 | 13 | 46 | | 39 | | 6 | | 104 | 269 |
|  | 8 | 16 | 84 | 63 | 11 | 174 | 16 | 55 | | 32 | | 7 | | 110 | 284 |
|  | 9 | 21 | 107 | 64 | 5 | 197 | 27 | 66 | | 38 | | 5 | | 136 | 333 |
|  | 10 | 23 | 101 | 61 | 11 | 196 | 14 | 76 | | 35 | | 11 | | 136 | 332 |
|  | 11 | 30 | 137 | 68 | 7 | 242 | 21 | 83 | | 38 | | 19 | | 161 | 403 |
|  | 12 | 23 | 72 | 60 | 8 | 163 | 23 | 66 | | 27 | | 8 | | 124 | 287 |
| **Year** | 2000 | 372 | 1082 | 740 | 158 | 2352 | 316 | 669 | | 408 | | 70 | | 1463 | 3815 |
| **Month** | 1 | 35 | 80 | 56 | 12 | 183 | 22 | 48 | | 31 | | 3 | | 104 | 287 |
|  | 2 | 46 | 114 | 73 | 15 | 248 | 29 | 63 | | 32 | | 6 | | 130 | 378 |
|  | 3 | 54 | 98 | 74 | 13 | 239 | 38 | 85 | | 34 | | 8 | | 165 | 404 |
|  | 4 | 24 | 81 | 68 | 13 | 186 | 23 | 42 | | 32 | | 5 | | 102 | 288 |
|  | 5 | 35 | 92 | 78 | 13 | 218 | 34 | 50 | | 28 | | 11 | | 123 | 341 |
|  | 6 | 25 | 81 | 81 | 16 | 203 | 32 | 55 | | 44 | | 3 | | 134 | 337 |
|  | 7 | 20 | 59 | 53 | 17 | 149 | 23 | 46 | | 43 | | 6 | | 118 | 267 |
|  | 8 | 20 | 80 | 55 | 13 | 168 | 17 | 58 | | 36 | | 6 | | 117 | 285 |
|  | 9 | 30 | 104 | 52 | 11 | 197 | 20 | 49 | | 25 | | 1 | | 95 | 292 |
|  | 10 | 25 | 97 | 57 | 11 | 190 | 29 | 55 | | 44 | | 4 | | 132 | 322 |
|  | 11 | 22 | 113 | 50 | 13 | 198 | 17 | 63 | | 34 | | 6 | | 120 | 318 |
|  | 12 | 36 | 83 | 43 | 11 | 173 | 32 | 55 | | 25 | | 11 | | 123 | 296 |
| **Year** | 2001 | 372 | 1090 | 720 | 156 | 2338 | 260 | 682 | | 424 | | 90 | | 1456 | 3794 |
| **Month** | 1 | 33 | 109 | 66 | 16 | 224 | 35 | 66 | | 36 | | 6 | | 143 | 367 |
|  | 2 | 28 | 85 | 66 | 12 | 191 | 24 | 53 | | 37 | | 6 | | 120 | 311 |
|  | 3 | 38 | 113 | 77 | 13 | 241 | 33 | 73 | | 49 | | 7 | | 162 | 403 |
|  | 4 | 43 | 82 | 53 | 15 | 193 | 17 | 52 | | 35 | | 5 | | 109 | 302 |
|  | 5 | 27 | 80 | 70 | 20 | 197 | 29 | 66 | | 34 | | 6 | | 135 | 332 |
|  | 6 | 39 | 92 | 65 | 11 | 207 | 26 | 46 | | 29 | | 9 | | 110 | 317 |
|  | 7 | 21 | 51 | 62 | 10 | 144 | 11 | 42 | | 30 | | 12 | | 95 | 239 |
|  | 8 | 21 | 90 | 51 | 15 | 177 | 16 | 52 | | 36 | | 7 | | 111 | 288 |
|  | 9 | 31 | 84 | 38 | 9 | 162 | 17 | 58 | | 27 | | 5 | | 107 | 269 |
|  | 10 | 24 | 106 | 50 | 9 | 189 | 17 | 58 | | 43 | | 9 | | 127 | 316 |
|  | 11 | 36 | 115 | 68 | 15 | 234 | 18 | 72 | | 41 | | 11 | | 142 | 376 |
|  | 12 | 31 | 83 | 54 | 11 | 179 | 17 | 44 | | 27 | | 7 | | 95 | 274 |
| **Year** | 2002 | 350 | 1048 | 632 | 124 | 2154 | 227 | 583 | | 398 | | 85 | | 1293 | 3447 |
| **Month** | 1 | 32 | 110 | 57 | 11 | 210 | 21 | 59 | | 42 | | 12 | | 134 | 344 |
|  | 2 | 43 | 111 | 60 | 11 | 225 | 29 | 48 | | 27 | | 7 | | 111 | 336 |
|  | 3 | 39 | 81 | 58 | 11 | 189 | 29 | 56 | | 28 | | 11 | | 124 | 313 |
|  | 4 | 32 | 79 | 65 | 19 | 195 | 20 | 47 | | 43 | | 10 | | 120 | 315 |
|  | 5 | 39 | 102 | 54 | 10 | 205 | 22 | 63 | | 34 | | 8 | | 127 | 332 |
|  | 6 | 32 | 66 | 41 | 10 | 149 | 22 | 40 | | 26 | | 10 | | 98 | 247 |
|  | 7 | 21 | 70 | 54 | 10 | 155 | 6 | 42 | | 41 | | 4 | | 93 | 248 |
|  | 8 | 15 | 79 | 31 | 6 | 131 | 9 | 53 | | 38 | | 5 | | 105 | 236 |
|  | 9 | 26 | 75 | 64 | 5 | 170 | 16 | 42 | | 32 | | 2 | | 92 | 262 |
|  | 10 | 15 | 87 | 57 | 8 | 167 | 14 | 42 | | 28 | | 6 | | 90 | 257 |
|  | 11 | 25 | 95 | 43 | 14 | 177 | 19 | 47 | | 28 | | 5 | | 99 | 276 |
|  | 12 | 31 | 93 | 48 | 9 | 181 | 20 | 44 | | 31 | | 5 | | 100 | 281 |
| **Year** | 2003 | 349 | 934 | 672 | 157 | 2112 | 262 | 574 | | 381 | | 108 | | 1325 | 3437 |
| **Month** | 1 | 34 | 85 | 48 | 12 | 179 | 23 | 57 | | 25 | | 9 | | 114 | 293 |
|  | 2 | 41 | 74 | 66 | 6 | 187 | 31 | 47 | | 42 | | 9 | | 129 | 316 |
|  | 3 | 42 | 106 | 68 | 15 | 231 | 30 | 43 | | 46 | | 15 | | 134 | 365 |
|  | 4 | 27 | 86 | 62 | 21 | 196 | 22 | 52 | | 35 | | 8 | | 117 | 313 |
|  | 5 | 37 | 87 | 96 | 17 | 237 | 20 | 53 | | 34 | | 13 | | 120 | 357 |
|  | 6 | 30 | 60 | 67 | 15 | 172 | 30 | 48 | | 24 | | 12 | | 114 | 286 |
|  | 7 | 26 | 53 | 56 | 15 | 150 | 14 | 42 | | 34 | | 6 | | 96 | 246 |
|  | 8 | 17 | 72 | 44 | 7 | 140 | 11 | 49 | | 16 | | 12 | | 88 | 228 |
|  | 9 | 16 | 105 | 42 | 20 | 183 | 19 | 46 | | 35 | | 5 | | 105 | 288 |
|  | 10 | 25 | 73 | 39 | 13 | 150 | 25 | 51 | | 33 | | 5 | | 114 | 264 |
|  | 11 | 32 | 68 | 44 | 7 | 151 | 26 | 49 | | 23 | | 7 | | 105 | 256 |
|  | 12 | 22 | 65 | 40 | 9 | 136 | 11 | 37 | | 34 | | 7 | | 89 | 225 |
| **Year** | 2004 | 262 | 943 | 628 | 163 | 1996 | 195 | 504 | | 389 | | 89 | | 1177 | 3173 |
| **Month** | 1 | 25 | 82 | 53 | 18 | 178 | 26 | 56 | | 25 | | 6 | | 113 | 291 |
|  | 2 | 33 | 76 | 45 | 12 | 166 | 26 | 32 | | 29 | | 10 | | 97 | 263 |
|  | 3 | 24 | 87 | 78 | 22 | 211 | 28 | 41 | | 43 | | 10 | | 122 | 333 |
|  | 4 | 21 | 79 | 74 | 14 | 188 | 23 | 38 | | 49 | | 5 | | 115 | 303 |
|  | 5 | 24 | 49 | 45 | 10 | 128 | 14 | 33 | | 30 | | 9 | | 86 | 214 |
|  | 6 | 21 | 66 | 50 | 9 | 146 | 14 | 38 | | 28 | | 3 | | 83 | 229 |
|  | 7 | 10 | 70 | 44 | 20 | 144 | 6 | 35 | | 29 | | 7 | | 77 | 221 |
|  | 8 | 17 | 71 | 48 | 10 | 146 | 5 | 43 | | 29 | | 9 | | 86 | 232 |
|  | 9 | 24 | 91 | 54 | 10 | 179 | 16 | 44 | | 28 | | 7 | | 95 | 274 |
|  | 10 | 14 | 86 | 34 | 9 | 143 | 9 | 43 | | 37 | | 4 | | 93 | 236 |
|  | 11 | 22 | 91 | 46 | 16 | 175 | 14 | 53 | | 34 | | 13 | | 114 | 289 |
|  | 12 | 27 | 95 | 57 | 13 | 192 | 14 | 48 | | 28 | | 6 | | 96 | 288 |
| **Year** | 2005 | 316 | 1005 | 692 | 161 | 2174 | 238 | 601 | | 364 | | 93 | | 1296 | 3470 |
| **Month** | 1 | 25 | 84 | 50 | 18 | 177 | 19 | 45 | | 32 | | 10 | | 106 | 283 |
|  | 2 | 30 | 74 | 52 | 15 | 171 | 27 | 52 | | 22 | | 7 | | 108 | 279 |
|  | 3 | 35 | 103 | 66 | 17 | 221 | 23 | 49 | | 33 | | 8 | | 113 | 334 |
|  | 4 | 37 | 71 | 63 | 8 | 179 | 20 | 56 | | 36 | | 8 | | 120 | 299 |
|  | 5 | 22 | 74 | 70 | 13 | 179 | 26 | 63 | | 27 | | 8 | | 124 | 303 |
|  | 6 | 32 | 98 | 64 | 15 | 209 | 27 | 55 | | 40 | | 7 | | 129 | 338 |
|  | 7 | 14 | 54 | 44 | 13 | 125 | 9 | 56 | | 22 | | 7 | | 94 | 219 |
|  | 8 | 19 | 77 | 48 | 19 | 163 | 6 | 44 | | 39 | | 8 | | 97 | 260 |
|  | 9 | 24 | 69 | 58 | 14 | 165 | 14 | 36 | | 20 | | 9 | | 79 | 244 |
|  | 10 | 21 | 93 | 45 | 10 | 169 | 16 | 47 | | 32 | | 6 | | 101 | 270 |
|  | 11 | 32 | 113 | 77 | 8 | 230 | 24 | 53 | | 33 | | 8 | | 118 | 348 |
|  | 12 | 25 | 95 | 55 | 11 | 186 | 27 | 45 | | 28 | | 7 | | 107 | 293 |
| **Year** | 2006 | 292 | 1024 | 628 | 143 | 2087 | 251 | 584 | | 390 | | 91 | | 1316 | 3403 |
| **Month** | 1 | 41 | 113 | 55 | 9 | 218 | 41 | 58 | | 37 | | 22 | | 158 | 376 |
|  | 2 | 39 | 101 | 48 | 13 | 201 | 29 | 57 | | 35 | | 7 | | 128 | 329 |
|  | 3 | 34 | 101 | 70 | 18 | 223 | 32 | 47 | | 34 | | 9 | | 122 | 345 |
|  | 4 | 25 | 84 | 76 | 13 | 198 | 10 | 51 | | 35 | | 7 | | 103 | 301 |
|  | 5 | 26 | 83 | 52 | 17 | 178 | 15 | 49 | | 23 | | 9 | | 96 | 274 |
|  | 6 | 22 | 77 | 59 | 14 | 172 | 22 | 26 | | 39 | | 8 | | 95 | 267 |
|  | 7 | 7 | 66 | 52 | 10 | 135 | 9 | 37 | | 30 | | 3 | | 79 | 214 |
|  | 8 | 18 | 65 | 44 | 7 | 134 | 5 | 50 | | 39 | | 4 | | 98 | 232 |
|  | 9 | 22 | 66 | 28 | 10 | 126 | 22 | 37 | | 25 | | 7 | | 91 | 217 |
|  | 10 | 15 | 85 | 53 | 10 | 163 | 24 | 57 | | 33 | | 7 | | 121 | 284 |
|  | 11 | 23 | 90 | 52 | 11 | 176 | 23 | 62 | | 27 | | 5 | | 117 | 293 |
|  | 12 | 20 | 93 | 39 | 11 | 163 | 19 | 53 | | 33 | | 3 | | 108 | 271 |
| **Year** | 2007 | 296 | 949 | 722 | 199 | 2166 | 221 | 578 | | 352 | | 109 | | 1260 | 3426 |
| **Month** | 1 | 16 | 82 | 57 | 14 | 169 | 17 | 53 | | 26 | | 8 | | 104 | 273 |
|  | 2 | 37 | 77 | 68 | 13 | 195 | 23 | 49 | | 24 | | 9 | | 105 | 300 |
|  | 3 | 43 | 81 | 77 | 24 | 225 | 26 | 55 | | 37 | | 7 | | 125 | 350 |
|  | 4 | 21 | 83 | 77 | 15 | 196 | 23 | 55 | | 26 | | 13 | | 117 | 313 |
|  | 5 | 26 | 85 | 66 | 20 | 197 | 23 | 58 | | 31 | | 9 | | 121 | 318 |
|  | 6 | 25 | 82 | 79 | 19 | 205 | 19 | 44 | | 43 | | 9 | | 115 | 320 |
|  | 7 | 10 | 74 | 63 | 18 | 165 | 17 | 49 | | 36 | | 15 | | 117 | 282 |
|  | 8 | 20 | 79 | 48 | 17 | 164 | 11 | 42 | | 29 | | 8 | | 90 | 254 |
|  | 9 | 29 | 82 | 43 | 13 | 167 | 17 | 41 | | 18 | | 8 | | 84 | 251 |
|  | 10 | 7 | 89 | 64 | 20 | 180 | 10 | 46 | | 32 | | 7 | | 95 | 275 |
|  | 11 | 30 | 90 | 42 | 9 | 171 | 15 | 53 | | 29 | | 9 | | 106 | 277 |
|  | 12 | 32 | 45 | 38 | 17 | 132 | 20 | 33 | | 21 | | 7 | | 81 | 213 |
| **Year** | 2008 | 255 | 889 | 558 | 142 | 1844 | 204 | 484 | | 366 | | 99 | | 1153 | 2997 |
| **Month** | 1 | 21 | 99 | 65 | 10 | 195 | 26 | 40 | | 30 | | 6 | | 102 | 297 |
|  | 2 | 29 | 80 | 60 | 14 | 183 | 34 | 53 | | 27 | | 8 | | 122 | 305 |
|  | 3 | 28 | 69 | 45 | 13 | 155 | 23 | 40 | | 30 | | 5 | | 98 | 253 |
|  | 4 | 25 | 64 | 52 | 18 | 159 | 22 | 40 | | 34 | | 12 | | 108 | 267 |
|  | 5 | 30 | 72 | 48 | 7 | 157 | 15 | 35 | | 30 | | 9 | | 89 | 246 |
|  | 6 | 17 | 67 | 36 | 6 | 126 | 14 | 43 | | 25 | | 5 | | 87 | 213 |
|  | 7 | 8 | 79 | 39 | 7 | 133 | 9 | 37 | | 40 | | 6 | | 92 | 225 |
|  | 8 | 9 | 65 | 37 | 11 | 122 | 9 | 39 | | 27 | | 7 | | 82 | 204 |
|  | 9 | 22 | 70 | 52 | 15 | 159 | 11 | 52 | | 33 | | 13 | | 109 | 268 |
|  | 10 | 13 | 69 | 47 | 19 | 148 | 15 | 34 | | 33 | | 15 | | 97 | 245 |
|  | 11 | 20 | 82 | 39 | 8 | 149 | 15 | 31 | | 29 | | 5 | | 80 | 229 |
|  | 12 | 33 | 73 | 38 | 14 | 158 | 11 | 40 | | 28 | | 8 | | 87 | 245 |
| **Year** | 2009 | 246 | 847 | 642 | 163 | 1898 | 212 | 433 | | 349 | | 109 | | 1103 | 3001 |
| **Month** | 1 | 20 | 85 | 65 | 13 | 183 | 20 | 42 | | 19 | | 3 | | 84 | 267 |
|  | 2 | 25 | 72 | 53 | 16 | 166 | 33 | 37 | | 24 | | 14 | | 108 | 274 |
|  | 3 | 28 | 109 | 67 | 15 | 219 | 35 | 51 | | 42 | | 11 | | 139 | 358 |
|  | 4 | 21 | 70 | 77 | 19 | 187 | 14 | 36 | | 45 | | 10 | | 105 | 292 |
|  | 5 | 24 | 65 | 69 | 15 | 173 | 15 | 34 | | 34 | | 8 | | 91 | 264 |
|  | 6 | 25 | 64 | 72 | 16 | 177 | 17 | 35 | | 29 | | 7 | | 88 | 265 |
|  | 7 | 11 | 49 | 35 | 8 | 103 | 6 | 23 | | 33 | | 10 | | 72 | 175 |
|  | 8 | 11 | 66 | 30 | 14 | 121 | 7 | 24 | | 22 | | 6 | | 59 | 180 |
|  | 9 | 22 | 70 | 45 | 11 | 148 | 13 | 48 | | 30 | | 11 | | 102 | 250 |
|  | 10 | 17 | 78 | 52 | 14 | 161 | 13 | 39 | | 21 | | 9 | | 82 | 243 |
|  | 11 | 20 | 59 | 39 | 7 | 125 | 25 | 32 | | 24 | | 9 | | 90 | 215 |
|  | 12 | 22 | 60 | 38 | 15 | 135 | 14 | 32 | | 26 | | 11 | | 83 | 218 |
| **Year** | 2010 | 181 | 770 | 532 | 131 | 1614 | 164 | 401 | | 313 | | 89 | | 967 | 2581 |
| **Month** | 1 | 16 | 65 | 30 | 13 | 124 | 14 | 33 | | 22 | | 7 | | 76 | 200 |
|  | 2 | 22 | 75 | 43 | 8 | 148 | 17 | 35 | | 27 | | 4 | | 83 | 231 |
|  | 3 | 22 | 74 | 58 | 11 | 165 | 27 | 39 | | 16 | | 10 | | 92 | 257 |
|  | 4 | 16 | 71 | 47 | 15 | 149 | 14 | 35 | | 30 | | 9 | | 88 | 237 |
|  | 5 | 12 | 67 | 50 | 12 | 141 | 15 | 38 | | 29 | | 3 | | 85 | 226 |
|  | 6 | 16 | 65 | 61 | 14 | 156 | 17 | 31 | | 38 | | 16 | | 102 | 258 |
|  | 7 | 9 | 53 | 36 | 6 | 104 | 3 | 26 | | 32 | | 9 | | 70 | 174 |
|  | 8 | 14 | 49 | 41 | 14 | 118 | 11 | 30 | | 24 | | 4 | | 69 | 187 |
|  | 9 | 7 | 68 | 53 | 7 | 135 | 12 | 22 | | 25 | | 7 | | 66 | 201 |
|  | 10 | 16 | 42 | 40 | 18 | 116 | 14 | 39 | | 18 | | 6 | | 77 | 193 |
|  | 11 | 17 | 87 | 40 | 9 | 153 | 8 | 39 | | 23 | | 11 | | 81 | 234 |
|  | 12 | 14 | 54 | 33 | 4 | 105 | 12 | 34 | | 29 | | 3 | | 78 | 183 |
| **Year** | 2011 | 222 | 788 | 524 | 160 | 1694 | 166 | 381 | | 288 | | 99 | | 934 | 2628 |
| **Month** | 1 | 21 | 73 | 38 | 8 | 140 | 15 | 29 | | 29 | | 9 | | 82 | 222 |
|  | 2 | 23 | 67 | 51 | 15 | 156 | 22 | 37 | | 20 | | 11 | | 90 | 246 |
|  | 3 | 34 | 83 | 66 | 17 | 200 | 23 | 37 | | 23 | | 13 | | 96 | 296 |
|  | 4 | 9 | 54 | 44 | 20 | 127 | 15 | 39 | | 26 | | 8 | | 88 | 215 |
|  | 5 | 23 | 82 | 44 | 10 | 159 | 10 | 34 | | 27 | | 7 | | 78 | 237 |
|  | 6 | 20 | 55 | 43 | 12 | 130 | 15 | 23 | | 25 | | 11 | | 74 | 204 |
|  | 7 | 5 | 40 | 30 | 11 | 86 | 9 | 24 | | 22 | | 3 | | 58 | 144 |
|  | 8 | 9 | 67 | 49 | 13 | 138 | 5 | 40 | | 24 | | 11 | | 80 | 218 |
|  | 9 | 24 | 56 | 38 | 17 | 135 | 13 | 36 | | 24 | | 7 | | 80 | 215 |
|  | 10 | 18 | 65 | 58 | 11 | 152 | 15 | 23 | | 22 | | 4 | | 64 | 216 |
|  | 11 | 22 | 87 | 32 | 16 | 157 | 14 | 28 | | 28 | | 12 | | 82 | 239 |
|  | 12 | 14 | 59 | 31 | 10 | 114 | 10 | 31 | | 18 | | 3 | | 62 | 176 |
| **Year** | 2012 | 171 | 680 | 452 | 124 | 1427 | 156 | 338 | | 291 | | 86 | | 871 | 2298 |
| **Month** | 1 | 19 | 66 | 40 | 6 | 131 | 8 | 29 | | 23 | | 4 | | 64 | 195 |
|  | 2 | 22 | 68 | 30 | 13 | 133 | 17 | 24 | | 28 | | 9 | | 78 | 211 |
|  | 3 | 21 | 63 | 46 | 12 | 142 | 28 | 46 | | 26 | | 6 | | 106 | 248 |
|  | 4 | 8 | 51 | 48 | 10 | 117 | 12 | 31 | | 25 | | 8 | | 76 | 193 |
|  | 5 | 25 | 59 | 44 | 16 | 144 | 24 | 34 | | 25 | | 9 | | 92 | 236 |
|  | 6 | 13 | 44 | 40 | 9 | 106 | 16 | 20 | | 30 | | 7 | | 73 | 179 |
|  | 7 | 7 | 38 | 37 | 10 | 92 | 5 | 33 | | 21 | | 5 | | 64 | 156 |
|  | 8 | 12 | 57 | 39 | 12 | 120 | 7 | 24 | | 18 | | 12 | | 61 | 181 |
|  | 9 | 9 | 59 | 39 | 5 | 112 | 6 | 19 | | 26 | | 6 | | 57 | 169 |
|  | 10 | 10 | 68 | 41 | 12 | 131 | 6 | 21 | | 28 | | 7 | | 62 | 193 |
|  | 11 | 16 | 59 | 24 | 8 | 107 | 16 | 27 | | 23 | | 8 | | 74 | 181 |
|  | 12 | 9 | 48 | 24 | 11 | 92 | 11 | 30 | | 18 | | 5 | | 64 | 156 |
| **Grand Total** |  | 4874 | 15573 | 10382 | 2348 | 33177 | 3860 | 8971 | | 6077 | | 1496 | | 20404 | 53581 |

**Table 2: Positive Monospot test results by year, month age and gender**

|  |  | **Positive** |  |  |  |  |  |  |  |  |  |  |
| --- | --- | --- | --- | --- | --- | --- | --- | --- | --- | --- | --- | --- |
|  |  | **Female** |  |  |  |  | **Male** |  |  |  |  |  |
|  | **Age** | 0-14 | 15-24 | 25-44 | 45+ | Total | 0-14 | 15-24 | 25-44 | 45+ | Total | **Total** |
| **Year** | 1997 | 61 | 335 | 62 | 7 | 465 | 61 | 224 | 56 | 9 | 350 | 815 |
| **Month** | 1 | 3 | 36 | 4 |  | 43 | 4 | 25 | 2 | 2 | 33 | 76 |
|  | 2 | 6 | 34 | 8 |  | 48 | 5 | 24 | 8 |  | 37 | 85 |
|  | 3 | 3 | 42 | 7 | 1 | 53 | 4 | 23 | 3 |  | 30 | 83 |
|  | 4 | 7 | 26 | 4 | 1 | 38 | 11 | 21 | 2 |  | 34 | 72 |
|  | 5 | 9 | 38 | 7 |  | 54 | 4 | 19 | 6 |  | 29 | 83 |
|  | 6 | 5 | 13 | 6 | 1 | 25 | 5 | 15 | 2 | 1 | 23 | 48 |
|  | 7 | 5 | 17 | 5 | 2 | 29 | 4 | 6 | 6 |  | 16 | 45 |
|  | 8 | 3 | 16 | 5 | 1 | 25 | 3 | 14 | 4 |  | 21 | 46 |
|  | 9 | 3 | 25 | 7 |  | 35 | 2 | 15 | 1 | 1 | 19 | 54 |
|  | 10 | 5 | 23 | 3 | 1 | 32 | 5 | 21 | 7 | 1 | 34 | 66 |
|  | 11 | 4 | 38 | 3 |  | 45 | 6 | 15 | 11 | 3 | 35 | 80 |
|  | 12 | 8 | 27 | 3 |  | 38 | 8 | 26 | 4 | 1 | 39 | 77 |
| **Year** | 1998 | 70 | 332 | 65 | 20 | 487 | 54 | 279 | 69 | 19 | 421 | 908 |
| **Month** | 1 | 4 | 27 | 2 | 3 | 36 | 6 | 21 | 7 | 3 | 37 | 73 |
|  | 2 | 9 | 34 | 8 | 2 | 53 | 4 | 27 | 2 | 1 | 34 | 87 |
|  | 3 | 10 | 31 | 7 |  | 48 | 1 | 29 | 9 | 1 | 40 | 88 |
|  | 4 | 6 | 31 | 8 | 3 | 48 | 6 | 23 | 8 | 2 | 39 | 87 |
|  | 5 | 6 | 31 | 6 | 3 | 46 | 5 | 28 | 6 | 3 | 42 | 88 |
|  | 6 | 5 | 18 | 1 | 2 | 26 | 7 | 12 | 1 | 3 | 23 | 49 |
|  | 7 | 6 | 25 | 2 | 2 | 35 | 4 | 20 | 13 |  | 37 | 72 |
|  | 8 | 6 | 23 | 6 | 2 | 37 | 5 | 24 | 6 | 4 | 39 | 76 |
|  | 9 | 3 | 41 | 6 | 1 | 51 | 3 | 20 | 3 |  | 26 | 77 |
|  | 10 | 3 | 15 | 4 | 2 | 24 | 1 | 28 | 4 | 1 | 34 | 58 |
|  | 11 | 2 | 33 | 9 |  | 44 | 5 | 22 | 4 |  | 31 | 75 |
|  | 12 | 10 | 23 | 6 |  | 39 | 7 | 25 | 6 | 1 | 39 | 78 |
| **Year** | 1999 | 63 | 228 | 41 | 9 | 341 | 37 | 226 | 52 | 10 | 325 | 666 |
| **Month** | 1 | 9 | 15 | 7 |  | 31 | 4 | 22 | 5 |  | 31 | 62 |
|  | 2 | 11 | 26 | 4 | 2 | 43 | 10 | 26 | 9 |  | 45 | 88 |
|  | 3 | 6 | 33 | 5 | 1 | 45 | 3 | 20 | 10 |  | 33 | 78 |
|  | 4 | 7 | 30 | 2 | 3 | 42 | 3 | 21 | 2 |  | 26 | 68 |
|  | 5 | 8 | 15 | 3 | 1 | 27 | 3 | 21 | 4 | 2 | 30 | 57 |
|  | 6 | 3 | 19 | 3 |  | 25 | 1 | 17 | 5 | 1 | 24 | 49 |
|  | 7 | 1 | 10 | 1 |  | 12 |  | 17 | 4 |  | 21 | 33 |
|  | 8 | 1 | 14 | 1 | 2 | 18 | 2 | 14 | 3 | 2 | 21 | 39 |
|  | 9 | 4 | 17 | 4 |  | 25 | 2 | 21 | 3 | 4 | 30 | 55 |
|  | 10 | 1 | 23 | 5 |  | 29 |  | 12 | 5 |  | 17 | 46 |
|  | 11 | 7 | 18 | 3 |  | 28 | 4 | 20 |  |  | 24 | 52 |
|  | 12 | 5 | 8 | 3 |  | 16 | 5 | 15 | 2 | 1 | 23 | 39 |
| **Year** | 2000 | 43 | 227 | 47 | 6 | 323 | 29 | 206 | 43 | 5 | 283 | 606 |
| **Month** | 1 | 4 | 13 | 3 |  | 20 | 1 | 12 | 2 |  | 15 | 35 |
|  | 2 | 4 | 20 | 2 | 3 | 29 | 3 | 16 | 2 |  | 21 | 50 |
|  | 3 | 6 | 31 | 4 |  | 41 | 3 | 27 | 1 |  | 31 | 72 |
|  | 4 | 3 | 19 | 3 |  | 25 | 2 | 17 | 4 | 2 | 25 | 50 |
|  | 5 | 1 | 9 | 5 |  | 15 | 5 | 21 | 4 | 1 | 31 | 46 |
|  | 6 | 6 | 25 | 5 |  | 36 | 2 | 6 | 3 | 1 | 12 | 48 |
|  | 7 | 1 | 12 | 2 |  | 15 | 2 | 13 | 3 |  | 18 | 33 |
|  | 8 | 3 | 14 | 3 | 1 | 21 | 4 | 13 | 5 |  | 22 | 43 |
|  | 9 | 6 | 27 | 6 |  | 39 | 3 | 21 | 5 |  | 29 | 68 |
|  | 10 | 2 | 22 | 4 |  | 28 | 2 | 20 | 5 | 1 | 28 | 56 |
|  | 11 | 3 | 19 | 7 | 1 | 30 | 1 | 30 | 8 |  | 39 | 69 |
|  | 12 | 4 | 16 | 3 | 1 | 24 | 1 | 10 | 1 |  | 12 | 36 |
| **Year** | 2001 | 52 | 197 | 33 | 9 | 291 | 34 | 218 | 41 | 7 | 300 | 591 |
| **Month** | 1 | 5 | 20 | 3 | 1 | 29 | 3 | 19 | 2 |  | 24 | 53 |
|  | 2 | 5 | 12 | 4 | 1 | 22 | 4 | 18 | 1 |  | 23 | 45 |
|  | 3 | 6 | 24 | 3 | 2 | 35 | 4 | 16 | 6 | 1 | 27 | 62 |
|  | 4 | 7 | 18 | 3 | 1 | 29 | 1 | 16 | 4 |  | 21 | 50 |
|  | 5 | 8 | 16 | 4 |  | 28 | 3 | 18 | 3 |  | 24 | 52 |
|  | 6 | 4 | 11 | 1 |  | 16 | 5 | 20 |  | 1 | 26 | 42 |
|  | 7 | 2 | 9 | 4 | 1 | 16 | 2 | 11 | 2 | 2 | 17 | 33 |
|  | 8 | 6 | 16 | 2 | 1 | 25 | 1 | 21 | 5 |  | 27 | 52 |
|  | 9 | 1 | 20 | 2 | 1 | 24 | 4 | 14 | 2 | 1 | 21 | 45 |
|  | 10 | 4 | 22 | 3 |  | 29 | 4 | 22 | 9 | 1 | 36 | 65 |
|  | 11 | 4 | 15 | 2 | 1 | 22 | 2 | 28 | 5 | 1 | 36 | 58 |
|  | 12 |  | 14 | 2 |  | 16 | 1 | 15 | 2 |  | 18 | 34 |
| **Year** | 2002 | 47 | 229 | 32 | 4 | 312 | 25 | 169 | 43 | 3 | 240 | 552 |
| **Month** | 1 | 4 | 19 |  |  | 23 | 1 | 15 | 4 | 1 | 21 | 44 |
|  | 2 | 4 | 28 | 3 |  | 35 | 2 | 20 | 6 | 1 | 29 | 64 |
|  | 3 | 4 | 27 | 2 |  | 33 | 3 | 11 | 2 |  | 16 | 49 |
|  | 4 | 2 | 17 | 4 | 2 | 25 | 1 | 25 | 1 |  | 27 | 52 |
|  | 5 | 8 | 18 | 2 |  | 28 |  | 12 | 3 |  | 15 | 43 |
|  | 6 | 5 | 7 | 1 |  | 13 | 1 | 12 | 3 |  | 16 | 29 |
|  | 7 | 3 | 17 | 4 | 1 | 25 | 3 | 10 | 5 |  | 18 | 43 |
|  | 8 | 5 | 21 | 3 |  | 29 | 2 | 12 | 5 |  | 19 | 48 |
|  | 9 | 3 | 19 | 3 |  | 25 | 1 | 12 | 3 |  | 16 | 41 |
|  | 10 | 5 | 20 | 1 |  | 26 | 1 | 14 | 4 | 1 | 20 | 46 |
|  | 11 | 2 | 17 | 3 |  | 22 | 6 | 14 | 4 |  | 24 | 46 |
|  | 12 | 2 | 19 | 6 | 1 | 28 | 4 | 12 | 3 |  | 19 | 47 |
| **Year** | 2003 | 47 | 195 | 34 | 6 | 282 | 38 | 181 | 40 | 11 | 270 | 552 |
| **Month** | 1 | 5 | 17 | 1 | 1 | 24 | 4 | 15 | 4 |  | 23 | 47 |
|  | 2 | 5 | 12 | 2 | 1 | 20 | 2 | 13 |  | 1 | 16 | 36 |
|  | 3 | 6 | 22 | 4 |  | 32 | 5 | 22 | 4 |  | 31 | 63 |
|  | 4 | 5 | 16 | 3 | 2 | 26 | 4 | 19 | 2 | 2 | 27 | 53 |
|  | 5 | 6 | 29 | 3 |  | 38 | 3 | 16 | 5 |  | 24 | 62 |
|  | 6 | 5 | 13 | 4 |  | 22 | 3 | 10 | 3 | 1 | 17 | 39 |
|  | 7 | 7 | 8 | 2 |  | 17 | 3 | 13 | 1 | 2 | 19 | 36 |
|  | 8 |  | 17 | 6 |  | 23 | 2 | 15 | 4 | 1 | 22 | 45 |
|  | 9 | 5 | 8 | 2 |  | 15 | 1 | 13 | 4 | 1 | 19 | 34 |
|  | 10 | 2 | 16 | 3 |  | 21 | 6 | 11 | 3 | 1 | 21 | 42 |
|  | 11 | 1 | 17 | 3 | 2 | 23 | 1 | 13 | 5 | 1 | 20 | 43 |
|  | 12 |  | 20 | 1 |  | 21 | 4 | 21 | 5 | 1 | 31 | 52 |
| **Year** | 2004 | 62 | 234 | 40 | 7 | 343 | 23 | 169 | 34 | 8 | 234 | 577 |
| **Month** | 1 | 7 | 22 | 3 |  | 32 | 3 | 17 | 2 | 1 | 23 | 55 |
|  | 2 | 9 | 24 | 5 |  | 38 | 2 | 17 | 4 | 1 | 24 | 62 |
|  | 3 | 5 | 27 | 6 |  | 38 |  | 18 | 4 |  | 22 | 60 |
|  | 4 | 4 | 24 | 1 |  | 29 | 5 | 8 | 2 |  | 15 | 44 |
|  | 5 | 4 | 21 | 3 |  | 28 | 1 | 14 | 2 |  | 17 | 45 |
|  | 6 | 9 | 14 | 9 | 3 | 35 | 3 | 13 | 3 | 1 | 20 | 55 |
|  | 7 | 1 | 15 | 3 | 2 | 21 | 4 | 17 | 3 | 1 | 25 | 46 |
|  | 8 | 4 | 19 | 3 | 1 | 27 | 1 | 13 | 1 | 3 | 18 | 45 |
|  | 9 | 4 | 10 | 2 | 1 | 17 | 2 | 10 | 5 |  | 17 | 34 |
|  | 10 | 4 | 10 | 3 |  | 17 |  | 12 | 3 |  | 15 | 32 |
|  | 11 | 3 | 25 |  |  | 28 | 1 | 14 | 1 |  | 16 | 44 |
|  | 12 | 8 | 23 | 2 |  | 33 | 1 | 16 | 4 | 1 | 22 | 55 |
| **Year** | 2005 | 38 | 171 | 25 | 2 | 236 | 24 | 174 | 28 | 11 | 237 | 473 |
| **Month** | 1 | 2 | 15 | 1 | 1 | 19 |  | 10 | 4 | 1 | 15 | 34 |
|  | 2 | 1 | 12 | 3 |  | 16 | 2 | 19 | 4 | 1 | 26 | 42 |
|  | 3 | 4 | 16 | 2 |  | 22 | 6 | 22 | 1 |  | 29 | 51 |
|  | 4 | 5 | 19 | 3 | 1 | 28 | 1 | 10 | 2 |  | 13 | 41 |
|  | 5 | 8 | 16 |  |  | 24 | 4 | 15 | 2 |  | 21 | 45 |
|  | 6 | 4 | 13 | 5 |  | 22 | 2 | 21 |  | 1 | 24 | 46 |
|  | 7 | 3 | 6 | 2 |  | 11 | 1 | 10 | 2 | 2 | 15 | 26 |
|  | 8 |  | 12 | 2 |  | 14 | 2 | 13 | 3 | 4 | 22 | 36 |
|  | 9 | 4 | 8 | 1 |  | 13 | 3 | 12 | 3 |  | 18 | 31 |
|  | 10 |  | 18 | 4 |  | 22 | 1 | 15 | 1 | 2 | 19 | 41 |
|  | 11 | 4 | 18 | 2 |  | 24 | 1 | 20 | 3 |  | 24 | 48 |
|  | 12 | 3 | 18 |  |  | 21 | 1 | 7 | 3 |  | 11 | 32 |
| **Year** | 2006 | 37 | 176 | 28 | 4 | 245 | 24 | 174 | 25 | 8 | 231 | 476 |
| **Month** | 1 | 4 | 15 | 1 |  | 20 | 3 | 15 | 2 |  | 20 | 40 |
|  | 2 | 2 | 9 | 4 |  | 15 | 2 | 10 |  | 1 | 13 | 28 |
|  | 3 | 4 | 13 | 3 | 1 | 21 | 3 | 8 | 1 | 2 | 14 | 35 |
|  | 4 |  | 13 |  |  | 13 | 3 | 15 | 1 |  | 19 | 32 |
|  | 5 | 4 | 14 | 2 | 1 | 21 | 2 | 15 | 3 |  | 20 | 41 |
|  | 6 | 7 | 10 | 2 |  | 19 | 3 | 22 | 1 |  | 26 | 45 |
|  | 7 | 3 | 10 | 3 |  | 16 | 3 | 9 | 5 |  | 17 | 33 |
|  | 8 | 1 | 21 | 3 |  | 25 | 1 | 21 |  | 1 | 23 | 48 |
|  | 9 | 3 | 10 |  | 1 | 14 |  | 17 | 1 | 2 | 20 | 34 |
|  | 10 | 3 | 22 | 3 |  | 28 | 2 | 14 | 4 |  | 20 | 48 |
|  | 11 | 5 | 25 | 6 | 1 | 37 |  | 14 | 2 | 1 | 17 | 54 |
|  | 12 | 1 | 14 | 1 |  | 16 | 2 | 14 | 5 | 1 | 22 | 38 |
| **Year** | 2007 | 35 | 165 | 20 | 5 | 225 | 28 | 151 | 33 | 5 | 217 | 442 |
| **Month** | 1 | 2 | 17 |  |  | 19 | 2 | 15 | 2 |  | 19 | 38 |
|  | 2 | 1 | 16 | 1 | 1 | 19 | 3 | 8 | 3 | 1 | 15 | 34 |
|  | 3 | 5 | 18 | 3 |  | 26 | 2 | 16 | 3 |  | 21 | 47 |
|  | 4 | 5 | 13 | 2 | 1 | 21 | 2 | 18 | 1 | 1 | 22 | 43 |
|  | 5 | 4 | 18 |  | 1 | 23 | 3 | 18 | 5 |  | 26 | 49 |
|  | 6 | 3 | 13 | 2 | 1 | 19 | 8 | 13 | 8 |  | 29 | 48 |
|  | 7 | 2 | 10 | 1 | 1 | 14 | 2 | 14 | 2 | 1 | 19 | 33 |
|  | 8 | 2 | 11 | 2 |  | 15 | 1 | 10 |  | 1 | 12 | 27 |
|  | 9 | 2 | 9 |  |  | 11 | 1 | 6 | 1 |  | 8 | 19 |
|  | 10 | 4 | 18 | 5 |  | 27 | 1 | 11 | 5 |  | 17 | 44 |
|  | 11 | 2 | 13 | 1 |  | 16 | 2 | 13 | 3 |  | 18 | 34 |
|  | 12 | 3 | 9 | 3 |  | 15 | 1 | 9 |  | 1 | 11 | 26 |
| **Year** | 2008 | 32 | 185 | 32 | 5 | 254 | 20 | 153 | 25 | 3 | 201 | 455 |
| **Month** | 1 | 4 | 18 | 4 |  | 26 | 1 | 10 | 2 |  | 13 | 39 |
|  | 2 | 5 | 20 | 5 | 1 | 31 | 6 | 11 | 1 |  | 18 | 49 |
|  | 3 | 3 | 16 | 3 |  | 22 | 2 | 14 |  | 1 | 17 | 39 |
|  | 4 | 3 | 15 | 2 |  | 20 | 2 | 17 | 1 |  | 20 | 40 |
|  | 5 | 2 | 17 | 2 |  | 21 | 1 | 11 | 5 | 1 | 18 | 39 |
|  | 6 | 3 | 14 | 5 |  | 22 |  | 20 | 1 | 1 | 22 | 44 |
|  | 7 | 2 | 12 | 2 |  | 16 | 3 | 12 | 3 |  | 18 | 34 |
|  | 8 | 1 | 4 | 1 |  | 6 | 2 | 9 | 2 |  | 13 | 19 |
|  | 9 | 1 | 23 | 3 |  | 27 |  | 14 | 4 |  | 18 | 45 |
|  | 10 | 2 | 19 | 2 | 1 | 24 |  | 18 | 1 |  | 19 | 43 |
|  | 11 | 4 | 10 | 3 | 2 | 19 | 2 | 9 |  |  | 11 | 30 |
|  | 12 | 2 | 17 |  | 1 | 20 | 1 | 8 | 5 |  | 14 | 34 |
| **Year** | 2009 | 32 | 162 | 21 | 8 | 223 | 17 | 145 | 24 | 6 | 192 | 415 |
| **Month** | 1 | 3 | 7 |  | 2 | 12 | 3 | 16 | 1 |  | 20 | 32 |
|  | 2 | 2 | 7 | 2 |  | 11 | 1 | 10 | 1 | 1 | 13 | 24 |
|  | 3 | 6 | 18 | 1 |  | 25 | 4 | 7 | 2 | 1 | 14 | 39 |
|  | 4 | 3 | 20 | 2 | 1 | 26 |  | 10 |  |  | 10 | 36 |
|  | 5 |  | 12 | 1 | 1 | 14 | 1 | 14 | 2 | 2 | 19 | 33 |
|  | 6 |  | 14 | 2 |  | 16 | 3 | 12 | 4 |  | 19 | 35 |
|  | 7 | 4 | 16 | 1 | 1 | 22 |  | 9 | 3 |  | 12 | 34 |
|  | 8 | 2 | 12 | 2 |  | 16 |  | 10 | 1 |  | 11 | 27 |
|  | 9 | 7 | 17 | 1 |  | 25 | 1 | 8 | 3 |  | 12 | 37 |
|  | 10 | 1 | 9 | 6 | 2 | 18 | 1 | 13 | 1 |  | 15 | 33 |
|  | 11 | 1 | 15 | 3 |  | 19 | 1 | 15 | 2 | 1 | 19 | 38 |
|  | 12 | 3 | 15 |  | 1 | 19 | 2 | 21 | 4 | 1 | 28 | 47 |
| **Year** | 2010 | 34 | 178 | 29 | 5 | 246 | 13 | 113 | 29 | 2 | 157 | 403 |
| **Month** | 1 | 5 | 15 | 2 |  | 22 | 2 | 10 | 1 |  | 13 | 35 |
|  | 2 | 4 | 15 | 2 |  | 21 | 2 | 8 | 1 |  | 11 | 32 |
|  | 3 | 6 | 26 | 4 |  | 36 | 1 | 11 | 5 |  | 17 | 53 |
|  | 4 | 5 | 16 | 5 |  | 26 | 1 | 12 | 2 | 1 | 16 | 42 |
|  | 5 | 2 | 17 | 1 | 1 | 21 | 2 | 10 | 2 |  | 14 | 35 |
|  | 6 | 2 | 16 | 2 |  | 20 | 2 | 8 | 1 |  | 11 | 31 |
|  | 7 |  | 12 | 2 | 1 | 15 |  | 6 | 4 |  | 10 | 25 |
|  | 8 |  | 9 | 6 | 1 | 16 | 1 | 5 | 4 |  | 10 | 26 |
|  | 9 | 4 | 8 | 1 |  | 13 |  | 8 | 3 | 1 | 12 | 25 |
|  | 10 | 1 | 14 |  |  | 15 | 1 | 11 |  |  | 12 | 27 |
|  | 11 | 4 | 15 | 3 |  | 22 | 1 | 11 | 4 |  | 16 | 38 |
|  | 12 | 1 | 15 | 1 | 2 | 19 |  | 13 | 2 |  | 15 | 34 |
| **Year** | 2011 | 16 | 163 | 26 | 3 | 208 | 24 | 136 | 30 | 8 | 198 | 406 |
| **Month** | 1 | 1 | 9 |  |  | 10 | 4 | 15 | 2 |  | 21 | 31 |
|  | 2 |  | 16 | 1 |  | 17 | 3 | 4 | 1 | 1 | 9 | 26 |
|  | 3 | 4 | 14 |  |  | 18 | 4 | 16 | 5 | 1 | 26 | 44 |
|  | 4 |  | 23 | 5 |  | 28 |  | 9 | 3 |  | 12 | 40 |
|  | 5 | 1 | 16 | 4 | 1 | 22 | 4 | 19 | 1 | 1 | 25 | 47 |
|  | 6 | 2 | 12 | 3 |  | 17 |  | 12 | 4 | 1 | 17 | 34 |
|  | 7 |  | 9 | 1 | 2 | 12 |  | 13 | 1 |  | 14 | 26 |
|  | 8 | 2 | 16 | 4 |  | 22 | 4 | 14 | 2 | 1 | 21 | 43 |
|  | 9 | 1 | 10 | 3 |  | 14 |  | 5 |  | 2 | 7 | 21 |
|  | 10 |  | 14 | 2 |  | 16 |  | 9 | 3 |  | 12 | 28 |
|  | 11 | 3 | 11 | 2 |  | 16 | 1 | 12 | 4 |  | 17 | 33 |
|  | 12 | 2 | 13 | 1 |  | 16 | 4 | 8 | 4 | 1 | 17 | 33 |
| **Year** | 2012 | 22 | 123 | 19 | 1 | 165 | 8 | 101 | 30 | 6 | 145 | 310 |
| **Month** | 1 |  | 10 | 1 |  | 11 |  | 6 | 4 |  | 10 | 21 |
|  | 2 | 4 | 8 | 4 |  | 16 |  | 13 | 3 |  | 16 | 32 |
|  | 3 | 5 | 10 | 1 |  | 16 | 2 | 16 | 2 |  | 20 | 36 |
|  | 4 | 2 | 10 | 4 |  | 16 | 1 | 5 | 3 |  | 9 | 25 |
|  | 5 | 3 | 12 | 1 |  | 16 | 1 | 8 |  | 2 | 11 | 27 |
|  | 6 | 2 | 10 | 1 | 1 | 14 |  | 4 | 5 | 1 | 10 | 24 |
|  | 7 | 1 | 13 | 1 |  | 15 | 1 | 7 | 4 |  | 12 | 27 |
|  | 8 | 1 | 7 | 1 |  | 9 |  | 7 | 2 |  | 9 | 18 |
|  | 9 | 1 | 13 |  |  | 14 | 1 | 10 | 2 |  | 13 | 27 |
|  | 10 | 1 | 14 | 2 |  | 17 | 1 | 6 | 3 |  | 10 | 27 |
|  | 11 | 1 | 11 | 1 |  | 13 |  | 12 |  | 1 | 13 | 26 |
|  | 12 | 1 | 5 | 2 |  | 8 | 1 | 7 | 2 | 2 | 12 | 20 |
| **Grand Total** |  | 691 | 3300 | 554 | 101 | 4646 | 459 | 2819 | 602 | 121 | 4001 | 8647 |

**Table 3: Population by year, age and gender from Grampian ISD data [27]**

| **Year** | **Gender** | **Age** | **Grampian ISD Population** |
| --- | --- | --- | --- |
| 1997 | Female | 0-14 | 48195 |
|  |  | 15-24 | 35247 |
|  |  | 25-44 | 83046 |
|  |  | >45 | 106392 |
|  | Male | 0-14 | 50750 |
|  |  | 15-24 | 36130 |
|  |  | 25-44 | 86478 |
|  |  | >45 | 94045 |
| 1998 | Female | 0-14 | 47997 |
|  |  | 15-24 | 35124 |
|  |  | 25-44 | 83229 |
|  |  | >45 | 107541 |
|  | Male | 0-14 | 50338 |
|  |  | 15-24 | 36235 |
|  |  | 25-44 | 86990 |
|  |  | >45 | 95804 |
| 1999 | Female | 0-14 | 47130 |
|  |  | 15-24 | 35078 |
|  |  | 25-44 | 83238 |
|  |  | >45 | 109679 |
|  | Male | 0-14 | 49449 |
|  |  | 15-24 | 36413 |
|  |  | 25-44 | 86976 |
|  |  | >45 | 98455 |
| 2000 | Female | 0-14 | 46563 |
|  |  | 15-24 | 34314 |
|  |  | 25-44 | 81759 |
|  |  | >45 | 109816 |
|  | Male | 0-14 | 19066 |
|  |  | 15-24 | 35774 |
|  |  | 25-44 | 85264 |
|  |  | >45 | 98955 |
| 2001 | Female | 0-14 | 45870 |
|  |  | 15-24 | 34396 |
|  |  | 25-44 | 80685 |
|  |  | >45 | 111195 |
|  | Male | 0-14 | 48398 |
|  |  | 15-24 | 35917 |
|  |  | 25-44 | 84044 |
|  |  | >45 | 100835 |
| 2002 | Female | 0-14 | 45269 |
|  |  | 15-24 | 34664 |
|  |  | 25-44 | 80019 |
|  |  | >45 | 112716 |
|  | Male | 0-14 | 47866 |
|  |  | 15-24 | 36068 |
|  |  | 25-44 | 83531 |
|  |  | >45 | 102790 |
| 2003 | Female | 0-14 | 44801 |
|  |  | 15-24 | 35271 |
|  |  | 25-44 | 79429 |
|  |  | >45 | 114369 |
|  | Male | 0-14 | 47255 |
|  |  | 15-24 | 36467 |
|  |  | 25-44 | 82566 |
|  |  | >45 | 104808 |
| 2004 | Female | 0-14 | 44368 |
|  |  | 15-24 | 35412 |
|  |  | 25-44 | 79194 |
|  |  | >45 | 116543 |
|  | Male | 0-14 | 46808 |
|  |  | 15-24 | 36285 |
|  |  | 25-44 | 82985 |
|  |  | >45 | 107385 |
| 2005 | Female | 0-14 | 43826 |
|  |  | 15-24 | 35553 |
|  |  | 25-44 | 79122 |
|  |  | >45 | 118287 |
|  | Male | 0-14 | 46231 |
|  |  | 15-24 | 36129 |
|  |  | 25-44 | 83329 |
|  |  | >45 | 109591 |
| 2006 | Female | 0-14 | 43338 |
|  |  | 15-24 | 35751 |
|  |  | 25-44 | 79335 |
|  |  | >45 | 120270 |
|  | Male | 0-14 | 45755 |
|  |  | 15-24 | 36453 |
|  |  | 25-44 | 83660 |
|  |  | >45 | 111588 |
| 2007 | Female | 0-14 | 43116 |
|  |  | 15-24 | 36321 |
|  |  | 25-44 | 80372 |
|  |  | >45 | 122127 |
|  | Male | 0-14 | 45886 |
|  |  | 15-24 | 36735 |
|  |  | 25-44 | 85059 |
|  |  | >45 | 113199 |
| 2008 | Female | 0-14 | 43114 |
|  |  | 15-24 | 36236 |
|  |  | 25-44 | 79951 |
|  |  | >45 | 123837 |
|  | Male | 0-14 | 46000 |
|  |  | 15-24 | 36517 |
|  |  | 25-44 | 85020 |
|  |  | >45 | 115140 |
| 2009 | Female | 0-14 | 43094 |
|  |  | 15-24 | 36895 |
|  |  | 25-44 | 79958 |
|  |  | >45 | 125651 |
|  | Male | 0-14 | 45921 |
|  |  | 15-24 | 36961 |
|  |  | 25-44 | 84915 |
|  |  | >45 | 117131 |
| 2010 | Female | 0-14 | 43094 |
|  |  | 15-24 | 36895 |
|  |  | 25-44 | 79958 |
|  |  | >45 | 125651 |
|  | Male | 0-14 | 45921 |
|  |  | 15-24 | 36961 |
|  |  | 25-44 | 84915 |
|  |  | >45 | 117131 |
| 2011 | Female | 0-14 | 43094 |
|  |  | 15-24 | 36895 |
|  |  | 25-44 | 79958 |
|  |  | >45 | 125651 |
|  | Male | 0-14 | 45921 |
|  |  | 15-24 | 36961 |
|  |  | 25-44 | 84915 |
|  |  | >45 | 117131 |
| 2012 | Female | 0-14 | 43094 |
|  |  | 15-24 | 36895 |
|  |  | 25-44 | 79958 |
|  |  | >45 | 125651 |
|  | Male | 0-14 | 45921 |
|  |  | 15-24 | 36961 |
|  |  | 25-44 | 84915 |
|  |  | >45 | 117131 |

**Table 4: Population for the June 2009 Scottish population by age and gender used for standardisation [29]**

| Gender | Age in years | Population |
| --- | --- | --- |
| Female | 0-14 | 43094 |
|  | 15-24 | 36895 |
|  | 25-44 | 79958 |
|  | >45 | 125651 |
| Male | 0-14 | 45921 |
|  | 15-24 | 36961 |
|  | 25-44 | 84915 |
|  | >45 | 117131 |
